# Assessing the feasibility of mechanical recycling for plastic tree shelters used in agriculture and forestry: degradation and contamination of waste

**DOI:** 10.1007/s11356-025-37021-y

**Published:** 2025-10-08

**Authors:** Ignacio Bernabé, Ma Ulagares de la Orden, Enrique Blázquez-Blázquez, Ma Luisa Cerrada, Gabriela Castro, Victoria Fernández-Fernández, Miguel Cobo-Golpe, María Ramil, Isaac Rodríguez, Joaquín Martínez Urreaga

**Affiliations:** 1 Department of Industrial and Environmental Chemical Engineering, E.T.S.I. Industriales, Universidad Politécnica de Madrid, Madrid, Spain; 2 Department of Organic Chemistry, Facultad de Óptica y Optometría, Universidad Complutense de Madrid, Madrid, Spain; 3 Institute of Science and Technology of Polymers (ICTP-CSIC), Madrid, Spain; 4 Department of Analytical Chemistry, Nutrition and Food Sciences, Aquatic One Health Research Center (ARCUS), Universidade de Santiago de Compostela, Santiago de Compostela, 15782 Spain

**Keywords:** Plastic tree shelters, Agriculture, Forestry, Contamination, Degradation, Mechanical recycling, Polypropylene

## Abstract

Plastic tree shelters are widely used in agriculture and forestry to protect young plants from predators and generate a favorable microclimate, thus increasing survival and growth rates. Considering that several thousand tons of plastic tree shelters (mostly polypropylene, PP) are used each year in Europe and that discarded aged shelters can release microplastics, residues of additives, and even pesticides, the destination of this material after use is a matter of concern. Mechanical recycling is the best option for these residues, but its technical feasibility depends on the polymer degradation level and its contamination, both by inorganic materials and by residues of additives and/or agrochemicals. Therefore, not all tree shelter waste is suitable for recycling. The main objective of this work is to characterize the degradation and contamination of PP tree shelters used in agriculture and forestry, to obtain information on the feasibility of their mechanical recycling. The results show the presence of fungicide residues only in some tubes used in agriculture. Although the external faces of the tubes appear strongly degraded, the degradation of the overall tube material is much less due to a dilution effect. Thus, we can conclude that most PP tree shelters, even those used for several years in harsh climates, could be successfully subjected to mechanical recycling.

**Supplementary Information:**

The online version contains supplementary material available at 10.1007/s11356-025-37021-y.

## Introduction

Tree shelters (or tube shelters or tree guards) are simple and economical devices that are widely used in plantations, both in forestry and agriculture, for example, in vineyards, pistachio, or olive tree plantations. Their mission is multiple. On the one hand, they protect young plants from predators and aggressive agrochemical treatments and agronomic practices, and on the other hand, they generate a microclimate (temperature, relative humidity, and radiation) more suitable for the development of plants in certain climatic conditions (Martínez Urreaga et al. 2020; Yagi 2022; Thyroff et al. 2022). Therefore, in many cases, protectors allow a significant increase in survival and growth rates in plantations (Abe 2022). Shelters made from materials such as wood, cardboard, or metal are used in some applications, but those made from polymers, such as polyethylene (PE), polypropylene (PP), or different biobased polymers, play a main role in agriculture and forestry. Among the polymers used, PP stands out for its low cost, lightness, light transmission, and mechanical properties.

Although there is no unified and reliable data on the volume of use of these plastic shelters, it can be said, based on estimates, that several thousand tons are used in Europe each year, so the fate of these tree shelters, after their use, is a matter of concern. It must be considered that the usefulness of the shelters disappears after a few years, when the plants are already of an adequate size. After this time, the shelters used in agriculture are usually removed. Although the exact data is not known, it seems that the main destinations of this waste are incineration and landfilling, also including unmanaged landfilling (Graf et al. 2022). The situation worsens in the case of shelters used in forestry, which are often abandoned directly in the field. Improper management of used shelters leads to significant social and environmental problems. In addition to a loss of resources, it must be taken into account that the abandoned shelters, like other agricultural plastics, release micro- and nanoplastics (Briassoulis 2023; Filipe et al. 2023), as well as additive residues. In the case of shelters used in agriculture, they can also release pesticide residues accumulated during crop treatments.

Various studies have shown that mechanical recycling is the best destination for different agricultural plastic wastes because it saves raw materials and energy and drastically reduces the environmental problems associated with the wastes (Picuno et al. 2012; Gu et al. 2017). The same can be said for PP shelter waste (Chau et al. 2021). However, as with other agricultural plastic wastes (Briassoulis et al. 2012, 2013), used shelters present two important issues, namely, degradation and contamination, that affect the quality of the recycled material and therefore the technical and economic feasibility of the entire mechanical recycling process.

During the lifespan of the shelters, which can exceed 10 years, the polymer can undergo significant degradation due to the combined action of ultraviolet (UV) radiation, O_2_, heat, and other atmospheric agents, such as wind and rain, which promote degradation by eroding the plastic. In some cases, the products and processes used in crop treatments can also degrade the polymer. This degradation is critical because it generally reduces polymer properties (Rabello and White 1997a; Ojeda et al. 2011; Lv et al. 2015; Gijsman and Fiorio 2023; Prior et al. 2023), leading to low-performance recycled materials, which can make mechanical recycling of very degraded shelters unfeasible in some extreme cases.

Photooxidative degradation of PP has been widely studied, including some studies devoted to its natural weathering (Ojeda et al. 2011; Lv et al. 2015; Soccalingame et al. 2016; Xiong et al. 2017; Grause et al. 2020). The main consequences of photooxidation are chain scissions and the formation of unsaturated and oxidized compounds, all of which lead to severe changes in the polymer structure (Xiong et al. 2017) and a decrease in the performance of the material.

On the other hand, end-of-life shelters may accumulate a wide range of contaminants during their use in the field, including inorganic substances, biological contamination, additives, and their degradation products. Moreover, shelters used in agriculture may also contain traces of some fungicides and other pesticides used in crop treatments, which can diffuse into the plastic. Some of these substances are resistant to standard recycling processes and therefore can be present in the recycled plastic. The presence of these substances in recycled plastics constitutes an important issue because they may include, in some cases, potentially hazardous substances that end up in the recycled goods and because, in general, contaminants alter the properties and quality of recycled plastic (Nerín and Batlle 1999; Camacho and Karlsson 2001; Horodytska et al. 2020; Gall et al. 2021b; Chibwe et al. 2023; Cobo-Golpe et al. 2024). The presence of pesticides is also relevant for the degradation of agricultural plastics, since some of them promote degradation by reacting with stabilizing additives and eliminating them (Espí et al. 2007; Briassoulis et al. 2018).

Inorganic contamination in plastic waste can come from additives intentionally added to virgin plastic, such as the inorganic pigments frequently used in shelters, and from substances unintentionally incorporated during the plastic’s useful life. Several authors have shown that inorganic contamination significantly worsens the mechanical properties of different thermoplastics, for example, reducing tensile strength and elongation at break (Ghasemi et al. 2015; Horodytska et al. 2018; Gall et al. 2021a, b; Prior et al. 2023). In addition to inorganic contamination, the use of tubes in the ground can lead to the presence of contamination of biological origin, due to the presence of bacteria and other organisms that can cause the formation of biofilm and different deposits on the surface of the tube. Pietrelli has shown the formation of this type of deposit on PP used in nets for mussel farming (Pietrelli 2022). Likewise, Potrykus et al. (2021) have observed biological contamination on the surface of PP samples that had been deposited in landfills.

As for additives, antioxidants and light stabilizers are expected to have been used in the manufacturing of the shelters. While most of these additives will have been largely degraded over time, some traces of them may remain, together with their degradation products, in end-of-life plastics (Blázquez-Blázquez et al. 2020; Cobo-Golpe et al. 2024). Like other additives and contaminants, there is a possibility that some of these substances may be released during mechanical reprocessing of used shelters or may migrate from recycled material (Hahladakis et al. 2018). Therefore, the presence of these substances needs to be thoroughly examined.

The above considerations indicate that the study of the degradation and contamination of used tree shelters is of paramount importance to discuss the technical feasibility of their mechanical recycling. Therefore, the main objective of this work is to analyze the degradation and contamination of shelters used in Spain, both in agriculture and forestry, in order to gain information on the feasibility of their mechanical recycling, which would improve the sustainability of shelter use and reduce the environmental problems associated with their abandonment in the environment. If the degradation and contamination of the used tubes were moderate, the waste could be used, in various mixtures with virgin plastic, to obtain recycled plastics that could be used to replace virgin PP in applications such as the manufacture of automotive parts or furniture. A particularly interesting application for this recycled PP could be the production of new shelters for use in agriculture and forestry.

The research focused exclusively on shelters made with polypropylene, the most prevalent polymer for this application in Spain. Nevertheless, characterizing the degradation and contamination of used shelters remains a significant challenge because, in practice, various polymer grades with different additive formulations are used. Thus, a set of PP used tree shelters, including shelters used in vineyard plantations and others used in forestry and urban parks, was collected in various locations in Spain, with different climates. After washing, the shelters were ground and homogenized to obtain an actual image of the overall state of the polymer and not just the most exposed surfaces. The degradation of the polymer was studied by IR spectroscopy, thermal analysis, tensile testing, and measurements of melt flow rates (MFRs) and oxidation induction time (OIT). The inorganic contamination was characterized by gravimetry, optical microscopy, and Raman spectroscopy, and the presence of residues of additives and fungicides was analyzed by gas and liquid chromatography coupled to single and tandem mass spectrometry, respectively.

## Experimental section

### Materials

**Fig. 1:**
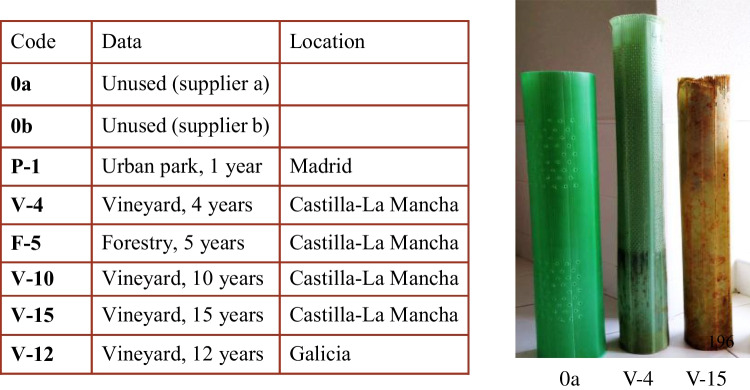
Code names and examples of the used tree shelters

Figure 1 shows the code names given to the tree shelters analyzed in this work, as well as an image of some of them. To obtain a representative set of the shelters used in Spain, tubes used during different times in various locations were collected. Madrid and Castilla-La Mancha are regions in the center of the country that are characterized by a continental climate with extreme temperatures, high sunshine, and moderate rainfall, while Galicia is located in the northwest of the country, in the Atlantic zone, and is characterized by a rainier climate, with fewer temperature changes and less sunshine than the center of the country. The selected shelters are all made of PP. Shelters used in vineyard plantations were included to take into account that the material used in agriculture may have been subjected to treatments with agrochemicals that can affect the quality of the waste. Since PP for shelters is not a standardized product and different polymer grades and additives are used, two types of unused shelters from different suppliers, with code names 0a and 0b, have been used as reference materials.

Analytical standards of pesticides were acquired from Sigma-Aldrich (Milwaukee, WI, USA) and Dr. Ehrenstorfer GmbH (Augsburg, Germany). Selection of target analytes was based on previous publications from the laboratory (Cobo-Golpe et al. 2024). Methanol (MeOH) of LC–MS grade was supplied by Merck (Darmstadt, Germany). Acetonitrile (ACN) and Triton X-100 were acquired from Thermo Fischer Scientifics (Waltham, MA, USA). Hexane (Hex), dichloromethane (DCM), and formic acid (FA) were purchased from VWR chemicals (Radnor, PA, USA). Ultrapure water (18.2 MΩ cm^−1^) was obtained using a Geni-U system (Rephile, Shanghai, China). Hydrophobic filters (0.22 μm pore size, 13 mm diameter) were purchased from Phenomenex (Torrance, CA, USA).

### Sample preparation

**Fig. 2:**
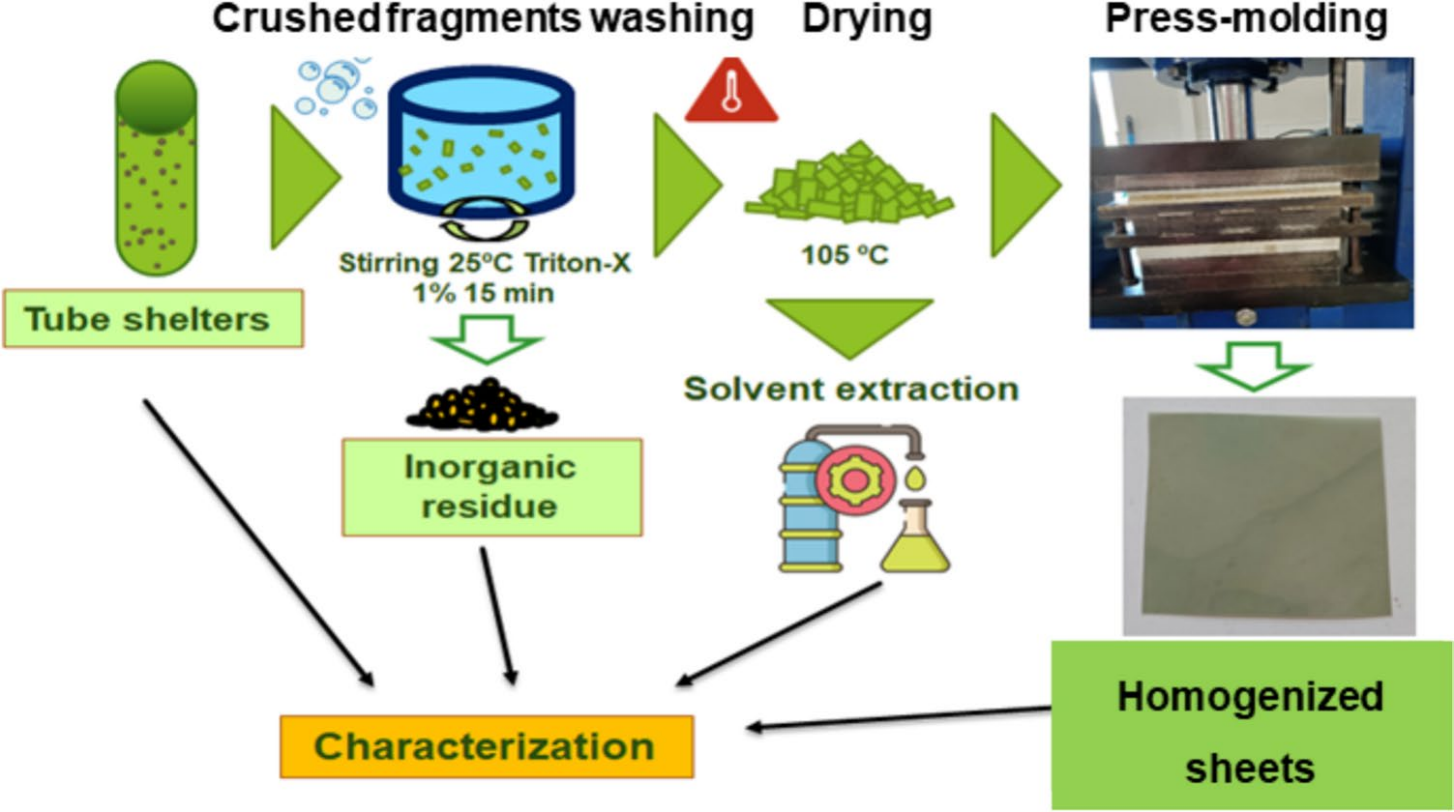
Scheme of sample preparation

After sampling, the collected tree shelters were cut and washed. At least three shelters were processed for each location and time of use. In order to simulate the usual waste washing conditions in the mechanical recycling of thermoplastics, our washing protocol involved stirring for 15 min at 25 °C in water with 1.0 wt. % of Triton X-100, a standard surfactant. After washing, the samples were dried at 105 °C in a vacuum oven. A part of the samples was crushed in a cryogenic mill using liquid nitrogen. Finally, homogenized sheets (thickness 200 ± 20 μm) were obtained by press-molding at 180 °C and 100 kg/cm^2^ in a IQAP LAP hydraulic press (IQAP, Spain). These homogenized sheets, representative of the shelters’ overall composition, provide data on the material’s properties relevant for recycling. Figure 2 shows a scheme of the procedure followed.

Since the double-walled tubes have two layers with differing exposure to atmospheric agents, some thermal and spectroscopic measurements were taken on the outer layer, as well as on the homogenized material, to compare the degradation levels of the more exposed parts with the overall shelter material.

Extraction of pesticides was performed according to Cobo-Golpe et al. (2024) with minor modifications. Briefly, 0.5 g of crushed material was spiked with 100 ng of a mixture of isotopically labeled surrogate standards (SSs). Then, 10 mL of DCM-to-Hex (50:50, v/v) was added to the sample and submitted to ultrasound-assisted extraction (UAE) for 15 min at 25 °C. The process was repeated twice, and the combined extract (≈20 mL) was concentrated to almost dryness under a gentle stream of nitrogen (N_2_) and reconstituted in 2 mL of MeOH-to-ACN (50:50, v/v). Finally, the obtained extract was filtered through a 0.22-µm hydrophobic filter prior to analysis by UHPLC-QqQ. Analysis of additive residues required a Soxhlet extraction with DCM for 8 h. The extract was concentrated to near dryness, first using a rotary evaporator, followed by the use of a gentle stream of nitrogen. The obtained extract was concentrated to near dryness under N_2_ and filled up to a final volume of 500 µL.

### Sample characterization

To evaluate the polymer degradation, the structure and properties of the materials were characterized by means of infrared spectroscopy (ATR-FTIR), solid colorimetry, thermogravimetric analysis (TGA), differential scanning calorimetry (DSC), determination of oxidation induction time (OIT) and melt flow rate (MFR), and tensile tests. Degradation tests were carried out on (a) the exposed outer part of the tubes, (b) their inner part, and (c) the homogenized sheets of each material.

The ATR-FTIR spectra were obtained using a Nicolet iS10 spectrometer equipped with a diamond attenuated total reflectance (ATR) accessory. The spectra (16 scans at a resolution of 4 cm^−1^) were normalized using the 1460 cm^−1^ band, assigned to a bending mode of CH_2_ groups in PP, as an internal reference (Lorenzo et al. 2011). The carbonyl index (CI) values were calculated as the ratio between the integrated band absorbance of the oxidation bands from 1850 to 1685 cm^−1^ and that of the methylene peak from 1500 to 1420 cm^−1^, as proposed by Almond et al. (2020).

Color was measured with a Minolta CM3600D Spectrocolorimeter, using a diffuse/8° geometry and including the specular reflectance. The instrument was calibrated against a white standard tile. The total color differences were calculated with the following equation:

1 $$\triangle\mathrm E^\ast=\left[\left(\triangle\mathrm L^\ast\right)^2+\left(\triangle\mathrm a^\ast\right)^2+\left(\triangle\mathrm b^\ast\right)^2\right]^{1/2}$$

where L^*^, a^*^, and b^*^ are the CIE (Commission Internationale de l´Eclairage) 1976 color space coordinates (de la Orden et al. 2007). A virgin green tube was used as a reference. Each color difference reported in this paper is the average of five measurements.

TGA was carried out with samples of 10–12 mg, in a TA Instruments TGA2050 thermobalance using 30 mL/min of dry nitrogen, between 40 and 800 °C at 10 K/min. DSC tests were performed in a TA Instruments DSC-Q20 calorimeter with a dry nitrogen flow of 50 mL/min and samples of 5–7 mg in standard aluminum pans. The following heating/cooling conditions were used: − 50 to 200 °C at 5 K/min, 3 min at 200 °C, 200 to − 50 °C at − 5 K/min, 3 min at − 50 °C, and − 50 to 200 °C at 5 K/min. The crystallinity (*X* (%)) of PP was determined using Eq. 1:

2 $$X=\frac{{\Delta H}_m}{{\Delta H}_0}\cdot100$$

where Δ*H*_*m*_ (J/g) is the melting enthalpy (second heating scan) of each sample and Δ*H*_0_ (J/g) is the melting enthalpy of a 100% crystalline PP sample, 207 J/g (Grebowicz et al. 1984).

OIT was analyzed in a TA Instruments 250 differential scanning calorimeter. Samples of about 5 mg were deposited in open aluminum pans, heated up at a rate of 20 °C/min until 190 °C under a nitrogen atmosphere, and maintained at that temperature for 3 min. Then, nitrogen was converted to oxygen, and the heat flow was recorded over time. The OIT value was estimated from the rise of the exothermic peak caused by oxygen absorption. The MFR values were measured at 230 °C with 2.16 kg, following the standard ISO 1133–2.

Tensile tests were performed following the standard ASTM D638, using a Shimadzu AGS-X.

testing machine, equipped with a 100 N load cell, with a crosshead speed of 10 mm/min. For each material, nine specimens were tested.

The nature of the inorganic contamination was studied by confocal microscopy and Raman microspectroscopy, using a Thermo Scientific™ DXR3 spectrometer (Thermo Fisher Scientific, Waltham, MA, USA) that includes an Olympus confocal microscope. The Raman scattering was excited using a laser at 532 nm. The spectra are the average of 32 scans, with a pinhole diameter of 25 μm. The resolution of the spectral shift was better than 3 cm^−1^. The amount of inorganic contamination was measured by calcination of three samples of each material for 60 min at 500 °C.

Pesticides (fungicides and insecticides) were determined by UPLC-MS/MS with an Acquity UPLC connected to a Xevo TQD triple quadrupole mass spectrometer (Waters, which contains a Z-spray ESI source). The LC column selected for the separation of the target analytes was a Zorbax Eclipse Plus C_18_ (2.1 × 50 mm, 1.8 µm) acquired from Agilent Technologies (Wilmington, DE, USA) and serially connected to a C_18_ 2.1 mm i.d. Security Guard™ cartridge obtained from Phenomenex (Torrance, CA, USA). Ultrapure water (A) and ACN (B), both containing 0.1% v/v of FA, were used as mobile phases at a constant flow rate of 0.4 mL min^−1^. The LC column and precolumn were maintained at 40 °C. The mobile phase gradient was programmed as follows: 2% B (0 min), 50% B (1.3–2.8 min), 100% B (6.4–7.5 min), and 2% B (7.6–10 min). The injection volume was 1 μL. Analytes were ionized under positive ionization mode (ESI +), except the fungicide Fludioxonil. N_2_ was employed as a gas at the ionization source (450 °C, 1000 L h^−1^). The capillary voltage was maintained at 1.5 kV and the cone voltage at 50 V. Data corresponding to determination conditions (retention times, precursor, quantification, and qualification ions for each compound, including their limits of quantification (LOQs)) are summarized in Table S1.

Analytical determination of additives was carried out using a Hewlett-Packard 6890 HRGC gas chromatograph equipped with an Agilent Technologies mass spectrometry detector (model 5973). The separation of the compounds was performed on a DB5-HT capillary column (15 m × 250 μm and 0.1 μm). The carrier gas used was helium with a flow rate of 1 mL min^−1^. The electronic impact (70 eV) was the type of ionization selected for the mass spectrometer. The chromatographic protocol was chosen according to previous investigations (Blázquez-Blázquez et al. 2019, 2020).

## Results and discussion

Characterization of the aged shelters has focused on two key factors that are fundamental for evaluating the technical feasibility of their mechanical recycling: (a) the degradation of the base polymer and (b) the presence of contamination, including both inorganic substances and residues of additives and pesticides.

### Degradation

PP in tree shelters can undergo significant photooxidative degradation during outdoor use, resulting in changes in the chemical nature and structure of the polymer, which, in turn, can lead to reduced performance of the resultant recycled material. Therefore, polymer degradation can question the feasibility and interest of the entire mechanical recycling process.

**Fig. 3:**
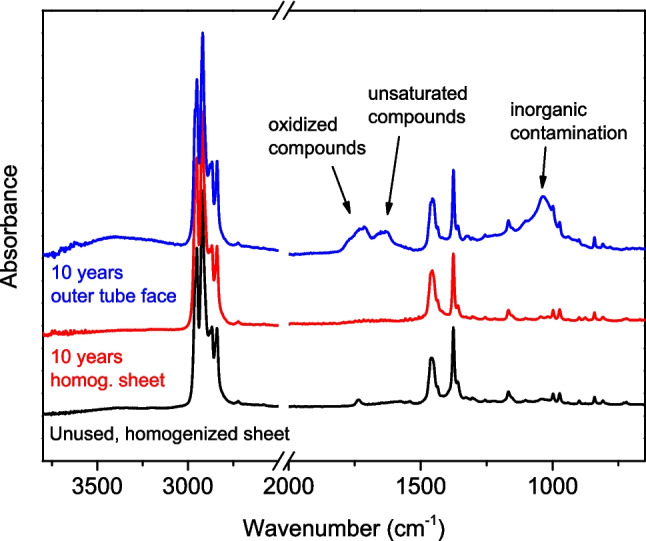
Normalized ATR-FTIR spectra of a tree shelter used for 10 years (outer shelter face and homogenized sheet) and a reference shelter

Changes in the chemical nature of the polymer have been studied using FTIR spectroscopy. Figure 3 shows the IR spectra of an unused shelter, which acts as the reference of non-degraded material, and of a shelter used for approximately 10 years in a vineyard plantation in Castilla-La Mancha. In the case of the aged material, both the spectrum of the outer face, which is the face most exposed to the sun and atmospheric agents, and that of the homogenized sheet are shown. This sheet was obtained from samples taken from the entire shelter and therefore indicates the actual state of the entire plastic.

The three spectra present the characteristic absorption bands of PP, which confirms that the base polymer of both shelters is PP. The appearance of a small absorption band at 720–730 cm^−1^ indicates that they are copolymers with ethylene. The two spectra of the used shelter are quite different from each other; while the spectrum of the homogenized sheet obtained from the tube is very similar to that of the reference shelter, the spectrum of the outer face shows strong absorptions that do not appear in the other spectra. The absorption at 1000–1120 cm^−1^, which can be assigned to silicates from the soil or introduced by the wind, shows the presence of inorganic contamination even after washing the residue. This contamination will be discussed later (the “Contamination” section).

Other new bands that appear in this spectrum can be assigned to products of the photooxidative degradation of PP. According to the accepted mechanisms for this type of degradation in polyolefins (Philippart et al. 1998; Lv et al. 2015; Soccalingame et al. 2016; Xiong et al. 2017; Wu et al. 2021), the unsaturated compounds generated during the degradation could contribute to the absorption bands observed at 1550–1680 cm^−1^. Likewise, hydroperoxides and other compounds with -OH groups could contribute to the broad band observed between 3000 and 3500 cm^−1^, and the carbonyl compounds formed in the degradation may contribute to the absorption bands observed between 1680 and 1800 cm^−1^. Although these bands can largely be explained by the photooxidative degradation, it is important to mention that other substances may also contribute to these absorptions. For example, the presence of water that could not be eliminated during the drying can contribute to the absorptions at 1550–1680 and 3000–3500 cm^−1^. On the other hand, the presence of substances of biological origin, adhered to the plastic as a result of microbial action during the prolonged contact with the soil, can also contribute to these absorptions, as Amide I and II bands appear in the 1550–1680 cm^−1^ region and the N–H stretching vibrations should appear between 3200 and 3400 cm^−1^.

It is important to emphasize that the characteristic bands of contamination and degradation appear in the spectrum of the outer face of the tube, but they are barely observed in the spectrum of the homogenized sheet of the same tube. To explain this result, it must be considered that the ATR spectra correspond to only a few microns of the material (1–2 µm with the experimental setup used in this work), so they report only the surface and not the whole material. The low intensity of the contamination and degradation bands in the homogenized sheet corresponds to a dilution phenomenon, which occurs when the entire shelter is crushed, mixed, and compression molded. A similar result was observed by Soccalingame et al. (2016), when they compared the ATR spectra recorded before and after reprocessing an UV-weathered PP composite. Overall, the spectra in Fig. 3 show that the contamination and degradation of the PP of the used tube are very moderate, even after 10 years of lifetime in this case, suggesting that its mechanical recycling could be feasible.

The different degradation of the surface and bulk of the material can also be observed in Fig. S-1, which shows the IR spectra taken at different points on a sample of a tube used for 10 years. The carbonyl index values measured at these points are also included. These results confirm that degradation is only significant on the exposed surfaces, especially the external one, and that it only minimally affects the material inside the tube, even after 10 years of use.

**Fig. 4:**
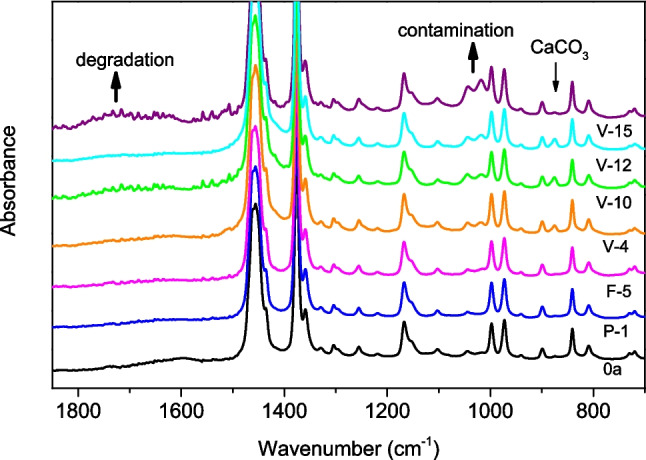
Normalized ATR-FTIR spectra of homogenized sheets of tree shelters used for different times in several locations and applications

As expected, degradation and contamination levels generally increase with the duration of shelter use, as shown in Fig. 4, which corresponds to homogenized sheets of different shelters, and S-2 (Supporting Information), which corresponds to the outer faces of the same shelters. However, both figures also indicate that this trend does not always exist; for instance, sample F-5, which has been used for 5 years, seems to show a lower level of degradation than sample V-4, which has been used for only 4 years. This apparent discrepancy can be explained by the differences in additive formulations and PP grades used during the shelter manufacturing. Furthermore, it must be considered that the degradation must depend not only on the time of use but also on the location and application. Shelters used in forestry, such as F-5, can be expected to be less exposed to UV light and agrochemicals than shelters used in agriculture, such as V-4. Regarding recyclability, Fig. 4 shows that the degradation of the whole polymer becomes evident after more than 10 years of use, which suggests that mechanical recycling may be poorly indicated in those cases.

The inorganic contamination observed in the IR spectra also depends on the location and type of use. Again, shelters employed in agriculture show higher levels of this type of contamination than those used in forestry. Figure 4 and Fig. S-2 also demonstrate that the nature of inorganic contamination depends on the location. For example, some shelters show bands at 835 and 1450 cm^−1^, indicating the presence of CaCO_3_ that is not observed in other tubes.

Degradation in use also leads to changes in the color of the material. Figure S-3 shows the appearance and color changes of the external and internal surfaces of tubes used for 4 and 10 years compared to a similar unused shelter. It is possible to observe that discoloration increases with time and is greater on the external surface, which is more exposed to the sun and other atmospheric agents.

Degradation has also been studied by thermal analysis (TG and DSC). Figure S-4 shows the TG curves of shelters used in different applications for different periods. The initial degradation temperature, which has been considered as the temperature at which 5% of the initial mass is lost (T5), is indicated on the graph by a dashed horizontal line. Although the mass loss curves are slightly different, as they correspond to shelters of different formulations that have been used in different applications, it can be observed that T5 decreases with time of use, but in all cases, it is greater than 300 °C. This result indicates that used PP shelters can be subjected to mechanical recycling, since they are susceptible to being reprocessed by extrusion under the usual conditions for PP.

DSC curves indicate, in addition to the degradation, the presence of different PP grades in the used shelters. Figure S-5 shows the curves corresponding to the second heating cycle in the two shelters selected as reference materials in this work. The two polymers show the characteristic melting endotherm of PP with melting temperatures (*T*_*m*_) between 160 and 170 °C, but the two *T*_*m*_ are clearly different. Furthermore, the reference material 0b shows a small melting endotherm at 123 °C, which seems to indicate the presence of PE in the form of a block copolymer with PP (Xu et al. 1997; Grigoriadi et al. 2024). This result agrees with the observation of characteristic PE bands in the infrared spectra. This endotherm at 123 °C does not appear in all the studied shelters and often is very weak, which indicates low PE content or the presence of random copolymers.

**Fig. 5:**
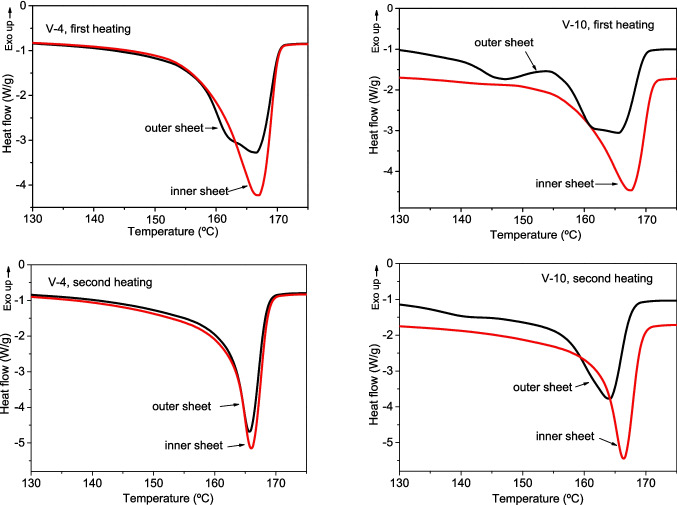
DSC curves (first and second heating scans) corresponding to tree shelters used for 4 (left) and 10 (right) years in vineyard plantations

The thermal analysis clearly shows the effect of photooxidative degradation. Figure 5 shows the DSC curves corresponding to the first and second heating scans of shelters used in vineyards in Castilla-La Mancha for 4 and 10 years. Since the tube shelters have a structure formed by two sheets, the exterior and interior sheets (with different exposure to atmospheric agents) of each shelter can be analyzed independently. As expected, the DSC curves of the first heating present severe degradation in the outer sheets, especially in the shelters used for 10 years.

Firstly, the outer sheet of the shelter used for 10 years shows a small endotherm at low temperature, *T*_*m*_ = 147 °C, which does not appear either on the inner sheet of the same tube, or in sheets of the shelter used for only 4 years. Secondly, the main melting endotherms of the outer sheets appear at lower temperatures and show a bimodal character that is not observed in the less exposed inner sheet and increases with the time of use of the shelter, revealing that it is a consequence of polymer degradation. These results indicate the presence of crystalline structures of lower regularity and then lower *T*_*m*_ in the aged shelters, which can be explained by the degradation and contamination of the outer face. Photooxidative degradation causes chain scission, leading to shorter chains that crystallize more easily, but also causes the appearance of new functional groups that act as irregularities or chemical defects, hampering crystallization and giving rise to less perfect crystalline structures with lower melting temperatures (Rabello and White 1997a; Tang et al. 2005; Lv et al. 2015; Xiong et al. 2017; Wu et al. 2021; Freudenthaler et al. 2023). Contamination introduced on the outside of the shelter during its use can also hinder crystallization.

To explain the bimodal character of the main melting endotherm, some authors have proposed that a fusion-recrystallization-fusion process may exist. Some crystals of low size and stability would melt first, crystallizing immediately into more stable structures that melt at higher temperatures (Rabello and White 1997b; Tang et al. 2005). Figure 5 shows the disappearance of the bimodal character of the main endotherm in the second heating, which supports the hypothesis of the existence of a melting-recrystallization-melting process in the first heating. The difference is also observed in the cooling scans (Fig. S-6). The crystalline structures that give rise to this process do not appear in the second heating because they are included in the main crystalline structures that are formed when the polymer crystallizes during cooling from the melt. The second heating curves show that the main melting endotherms of the outer sheets continue to appear at lower temperatures than those of the inner faces, although the difference is small in the 4-year-old shelter, because the crystals formed are thinner and less perfect and stable due to degradation and surface contamination. Furthermore, a second small endotherm is still observed at low temperature in the 10-year-old shelter, indicating the presence of small molecules that cannot be included in the main crystalline structures and form their own crystalline structures.

**Fig. 6:**
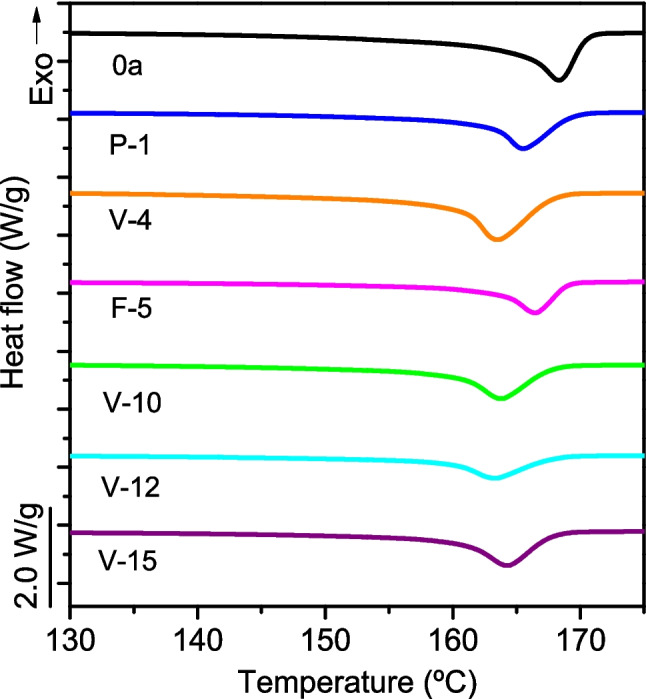
Second heating DSC curves corresponding to the homogenized sheets of tree shelters used in different applications over different periods

The effects of degradation and contamination are less evident when analyzing the homogenized sheets obtained from the whole shelter, due to the dilution effect that has already been observed in the IR spectra. The second heating curves of these homogenized sheets, shown in Fig. 6, do not reveal the low-temperature second melting endotherm observed in the external sheets. The main melting endotherms appear at lower temperatures in the used shelters than in the reference ones, but the differences observed are small, as can also be seen in the values of ∆*H*_*m*_ and *X* presented in Table S-2. The *T*_*m*_ values vary only between 163.3 and 168.3 °C. The results in Fig. 6 show that the effects of degradation and contamination in the thermal behavior of the material depend on the time of use and the type of application. In general, shelters used in agriculture show greater degradation than those used in forestry or similar, possibly due to greater exposure to sun and agrochemicals.

Table S-2 also displays the crystallinity values, determined from the melting enthalpy of the second heating scan, along with the crystallization temperature values. Polymers from different end-of-life shelters show similar crystallinity values, between 34 and 40%, and no clear trend has been observed in these values, which may be due to the existence of opposing effects. As previously mentioned, degradation can enhance the crystallization ability of the polymer by generating smaller chains. However, degradation and contamination can reduce crystallization ability by introducing impurities and chemical defects that hinder the crystallization of PP. The contamination, together with the chemical functionalities developed during the degradation, can also explain the decrease in the crystallization temperature of the polymer, from 129 to 122.2 °C, which is reported in Table S-2 and Figs. S-6 and S-7.

Results obtained in the thermal analysis are in good agreement with those obtained by IR spectroscopy. The thermal behavior of the polymers used demonstrates the effects of degradation and contamination, which generally depend on the time of use and the type of application of the shelter, as previously mentioned, but the degradation of the polymer is moderate if the whole shelter, and not only the external face, is considered.

**Fig. 7:**
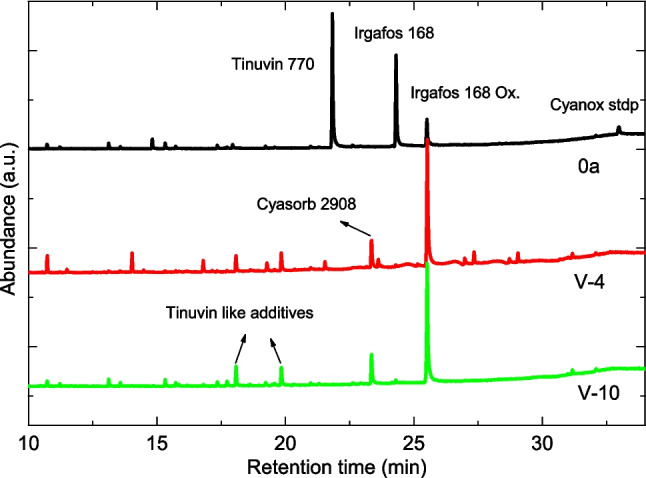
Gas chromatograms corresponding to homogenized samples 0a, V-4, and V-10

Thermal analysis has also been used to evaluate the OIT values. According to the obtained results, the used polymers present very low OIT values (Fig. S-8), i.e., a low resistance to oxidation, which may be related to the disappearance of antioxidant additives during the shelf lives of these PP shelters, as OIT values are highly dependent on the amount and type of antioxidants present in a given polymer (Blázquez-Blázquez et al. 2021). The presence of antioxidants and other additives has been studied by GC–MS. Figure 7 shows the chromatograms obtained for homogenized films of 0a, V-4, and V-10 items. Results exhibit differences between the compounds present in each material. The reference shelter (0a) is characterized by the presence of the UV protector Tinuvin 770 (a hindered amine light stabilizer) and the secondary antioxidant Irgafos 168. The polymers used during 4 and 10 years seem to have been produced with an additive formulation different from that of the 0a shelter. In V-4 and V-10, Cyasorb 2908 is the principal compound employed as an UV protection agent. Another remarkable fact that can be deduced from GC–MS records is that the secondary antioxidant Irgafos 168 has been massively consumed and converted into its oxidized form, Irgafos 168 Ox, before 4 years of weathering exposure. This result can explain the observed decrease in OIT values, confirming the need to add antioxidant additives during the mechanical recycling of the used tubes.

**Fig. 8:**
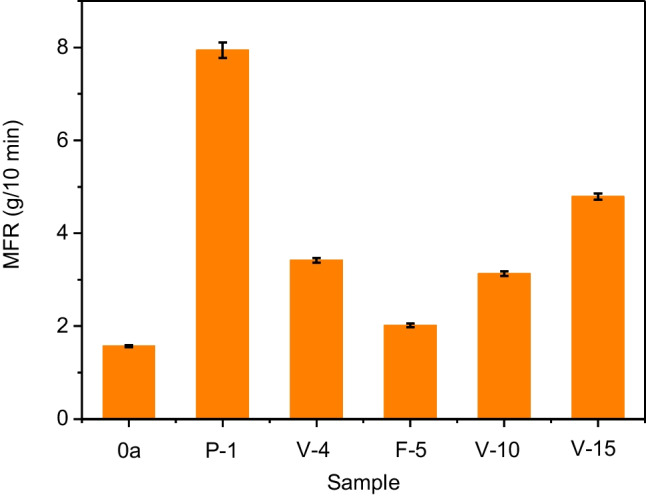
MFR values corresponding to crushed tree shelters

Polymer degradation in the aged shelters has also been characterized by tensile tests and measuring the MFR values, which play an important role in evaluating the processability of the recycled material. As expected, the MFR values reveal an increasing trend with the time of use (Fig. 8), which can be explained as a result of chain scission caused by photooxidative degradation. There are results that may seem anomalous, such as the high MFR value of the shelter used for only 1 year in an urban park in Madrid, but it must be considered that the collected shelters may correspond to different grades of PP. In any case, the MFR values achieved are moderate, so most of the degraded polymers could be processed under the usual PP conditions, especially if it is made in blends with virgin polymer or if viscosity control additives, such as peroxides, are used.

The results of the tensile tests are shown in Figure S-9. Firstly, it can be noted that the average values are usual for PP. The variability of the results is high, which may be due to the sample preparation procedure and the presence of inorganic impurities in the material. Regarding the trends shown by the results, it is observed that the elastic modulus hardly changes in the different materials and that both the tensile strength and the elongation at break are reasonably maintained during use, except for shelters used for 10 years or longer, in which the values decrease sharply. These results are in good agreement with those previously obtained through spectroscopy and thermal analysis and support the idea that shelters used, even for 10 years, show the structural characteristics, as well as the properties, that can make them a suitable resource for mechanical recycling. However, shelters used for longer periods show high degradation, which questions the technical feasibility of their mechanical recycling.

### Contamination

Taking into account that the studied shelters have been used in agriculture or forestry, it is expected that some of them present traces of the agrochemicals applied to crops, along with residues of additives used in the plastic formulation. Furthermore, they are likely to present inorganic contamination from environmental particles, since the shelters are partially buried in the ground and subjected to atmospheric elements such as rain and wind.

Inorganic contamination is relevant because it can alter some structural characteristics and properties of the recycled material, as previously shown, and it can reduce the lifespan of the equipment required for the mechanical recycling process, due to the abrasive character of some inorganic particles. In this work, the inorganic contamination of the end-of-life tubes has been evaluated by gravimetry, confocal optical microscopy, and Raman spectroscopy, in addition to FTIR-ATR spectroscopy, which, as shown in the “Degradation” section, reveals the presence of substances such as silicates and CaCO_3_ on the surface of some shelters.

**Fig. 9:**
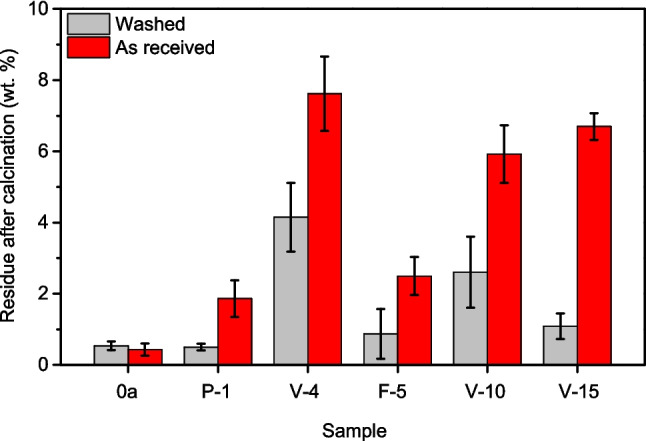
Calcination residues in used shelters before and after the washing process

Gravimetry allows us to estimate the amount of inorganic contamination in waste, as well as to evaluate the effectiveness of washing processes in removing that contamination. In this case, the used shelters were washed under conditions similar to those normally used in the recycling of thermoplastics. Before and after washing and drying, the samples were calcined in air to eliminate organic matter and obtain an estimate of the inorganic matter content. Figure 9 shows the residues after calcination, before and after the washing process. Generally, shelters used for a longer period contain a higher concentration of inorganic matter, although some samples exhibit an unusually high content, indicating that contamination is largely influenced by the location where tree shelters were placed. The washing process is effective, since it removes most of the inorganic contamination at a moderate cost. However, the results show that some of the contamination introduced during the service life cannot be removed by conventional washing.

**Fig. 10:**
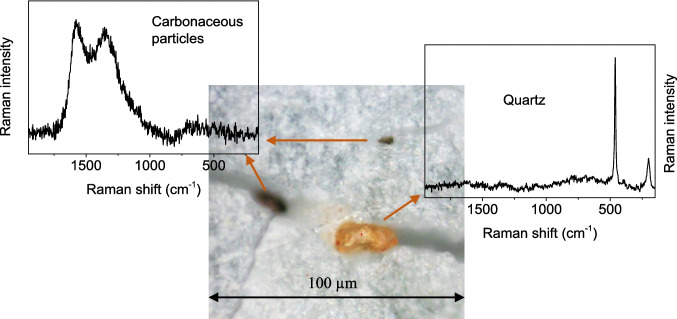
Characterization of contaminants in the outer surface of washed V-10

To assess the nature and location of wash-resistant inorganic pollutants, the shelter surfaces were studied using confocal optical microscopy and Raman spectroscopy. Figure 10 shows an area of the outer surface of a shelter used for 10 years in a vineyard in Castilla-La Mancha, after the washing process. Particles observed are embedded in the cracks generated on the surface of the plastic, as a result of its photooxidative degradation (Rabello and White 1997b; Xiong et al. 2017), which may explain the difficulty in removing them during the washing stage. Likewise, the encrustation of the particles in the cracks explains why the content of inorganic contamination generally increases with the time of use. Confocal Raman spectroscopy allows the analysis of particles of a few microns, revealing the presence of particles of many types on the tubes used in different locations. In the example presented in Fig. 10, Raman spectra showed the presence of the ubiquitous carbonaceous particles, identified by the characteristic D and G bands (Sze et al. 2001; Ferrugiari et al. 2015), as well as minerals such as quartz.

Contamination by pesticide residues is also a relevant concern because, if high, it can be a significant limitation for the recycling processes of the used shelters, as well as for the subsequent use of the recycled material.

UHPLC-MS/MS analysis demonstrated the absence of pesticide residues in new shelters, in samples collected from forest and urban parks, and in two out of four samples from agricultural fields at levels above LOQs (Table S1). However, sample V-10 contained low concentrations of two azolic fungicides, penconazole and myclobutanil (15.2 ng g^−1^ and 5.4 ng g^−1^, respectively). Both fungicides are considered relatively persistent compounds under environmental conditions (Pesticides Properties DataBase 2024) and have been previously reported in agricultural fields (Pérez-Mayán et al. 2020). In contrast, sample V12, which was used in Galicia, contained residues of ten pesticides (all of them employed as fungicides) above their LOQs (Table S3). Among them, the concentrations of dimethomorph (calculated as the sum of isomers) and cyprodinil exceeded 100 ng g^−1^. These compounds are used in the prevention and treatment of mildew and botrytis infections, respectively, which are more frequent in rainy regions. The relatively high fungicide concentrations in this shelter are in line with those previously reported for end-of-life agricultural plastics from the same geographic area (Cobo-Golpe et al. 2024). These results indicate that the presence of pesticide residues depends on both the type of application and the area in which the shelters are used. It should be expected that, in general, the residues from shelters used in forestry do not contain pesticide residues at significant levels and that residues from rainy areas contain higher levels of some fungicides than those from less rainy areas.

Finally, other substances that may appear in used shelters are residues of the additives originally incorporated into the polymer during the manufacturing process, such as antioxidants or UV absorbers. These residues include both additives that have not been consumed and reaction products formed over time. As commented in the “Degradation” section (Fig. 7), shelters used for 4 and 10 years still contain appreciable amounts of UV protection agents such as Cyasorb 2908 and Tinuvin-type compounds, together with the oxidized form of the antioxidant Irgafos 168. Knowledge of the presence of these additives in the formulation is key to ensuring an appropriate formulation of additives in the subsequent mechanical recycling.

## Conclusions

Polypropylene tree shelters used for different periods of time in various locations in Spain, both in agriculture and forestry, were studied to assess the technical feasibility of their mechanical recycling. The characterization focused on two fundamental aspects for recycling: the degradation of the polymer and the presence of pollutants, such as inorganic particles and residues of polymer additives and agrochemicals commonly applied in agriculture.

PP from used shelters shows changes in chemical composition and structural characteristics that can be expected in materials subjected to photooxidative degradation outdoors. Degradation depends on the climate and the application of the shelter, being higher in shelters used in agriculture than in those used in forestry, due to greater exposure to sun and wind, and the use of agrochemicals and more aggressive agronomic techniques. It should be noted that polymer degradation is very moderate when considering the whole shelter material and not just the most exposed exterior surface. For example, neither the elastic modulus nor the tensile strength shows significant differences in shelters used for even 10 years. The crystallinity of all the shelters studied varies only between 34 and 40%. PP degradation, which results in a worsening of some polymer properties, only limits the feasibility of mechanical recycling of shelters used for more than 10 years, as evidenced by the MFR values and mechanical properties.

Regarding the contamination of used shelters, it has been observed that simple washing processes, at room temperature, can eliminate most of the inorganic pollutants, mainly composed of silicates and CaCO_3_. Shelters also show the presence of residues of stabilizing additives that have not been completely consumed, such as Cyasorb 2908, as well as products generated from consumed additives, such as the oxidized form of Irgafos 168. The presence of pesticides (two azole fungicides) was observed in tubes used in agriculture in rainy areas.

In summary, the results obtained in this research indicate that most PP tree shelters used for 10 years or less could be suitable for collection and recovery through mechanical recycling. It would be interesting to study the properties of recycled materials obtained from these used shelters, mixed with virgin polypropylene in different proportions, as well as the viability of their use in different PP applications, such as the manufacture of new tree shelters.

## Supplementary Information

Below is the link to the electronic supplementary material.

ESM1 (DOCX 25.7 MB)

## Data Availability

Data is available upon reasonable request to the corresponding author.
