# Supplementary material for: Assessing the feasibility of mechanical recycling for plastic tree shelters used in agriculture and forestry: degradation and contamination of waste
Source: Environ Sci Pollut Res Int. 2025 Oct 8;32(40):23148–61. doi: 10.1007/s11356-025-37021-y (PMC12553568; doi:10.1007/s11356-025-37021-y)
Supplement: Supplementary file 1 — (DOCX 25.7 MB) [file 11356_2025_37021_MOESM1_ESM.docx]

**Supplementary material**

**Assessing the feasibility of mechanical recycling for plastic tree shelters used in agriculture and forestry: degradation and contamination of waste.**

I. Bernabé^*^, M.U. de la Orden, E. Blázquez-Blázquez, M. L. Cerrada, G. Castro, V. Fernández-Fernández, M. Cobo, M. Ramil, I. Rodríguez, J. Martínez Urreaga^*^

Author information

e-mail: [joaquin.martinez@upm.es](mailto:joaquin.martinez@upm.es)

Table S-1. Summary of investigated pesticides with LC-ESI-MS/MS determination parameters and limits of quantification (LOQs) in plastic residues.

| Use | **Compound** | **Ret. time (min)** | **Ion precursor** | **Cone voltage (V)** | **Q1 m/z (EC. eV)** | **Q2 m/z (EC. eV)** | **Ratio** **(Q2/Q1)** | **LOQ (ng g^-1^)** | **Surrogate Standard (SS)** |
| --- | --- | --- | --- | --- | --- | --- | --- | --- | --- |
| Insecticide | Acetamiprid | 2.32 | 223.0 | 34 | 126.0 (20) | 56.1 (15) | 0.55 | 2 | Acetamiprid-d_3_ |
| Fungicide | Ametroctadin | 3.49 | 276.0 | 60 | 176.0 (35) | 70.0 (50) | 0.23 | 4 | Myclobutanil-d_4_ |
| Fungicide | Azoxystrobin | 3.63 | 404.0 | 28 | 372.0 (15) | 329.0 (30) | 0.24 | 2 | Myclobutanil-d_4_ |
| Fungicide | Benalaxyl | 4.59 | 326.1 | 26 | 148.0 (20) | 91.0 (34) | 0.74 | 2 | Metalaxyl ^13^C_6_ |
| Fungicide | Boscalid | 3.99 | 342.9 | 41 | 139.9 (20) | 307.0 (20) | 1.9 | 4 | Boscalid-d_4_ |
| Fungicide | Bupirimate | 3.17 | 317.0 | 40 | 166.0 (25) | 108.0 (30) | 1.14 | 4 | Metalaxyl ^13^C_6_ |
| Fungicide | Carbendazim | 1.93 | 192.1 | 33 | 160.1 (18) | 132.1 (28) | 0.18 | 8 | Carbendazim-d_3_ |
| Insecticide | Chlorantraniliprole | 3.17 | 484.0 | 18 | 286.0 (12) | 453.0 (17) | 0.87 | 4 | Myclobutanil-d_4_ |
| Insecticide | Chlorpyrifos | 5.79 | 349.9 | 36 | 97.0 (32) | 198.0 (20) | 0.63 | 8 | Myclobutanil-d_4_ |
| Insecticide | Chlorpyrifos methyl | 5.08 | 321.8 | 34 | 125.0 (20) | 289.9 (16) | 0.37 | 20 | Myclobutanil-d_4_ |
| Fungicide | Clofentezine | 4.95 | 303.0 | 28 | 138.0 (22) | 102.0 (35) | 0.96 | 20 | Metalaxyl ^13^C_6_ |
| Insecticide | Clothianidin | 2.22 | 250.0 | 24 | 169.0 (22) | 132.0 (18) | 0.47 | 8 | Clothianidin-d_3_ |
| Fungicide | Cyflufenamid | 5.3 | 413.2 | 36 | 295.1 (15) | 203.0 (35) | 0.6 | 2 | Metalaxyl ^13^C_6_ |
| Fungicide | Cyproconazol | 3.36 | 292.2 | 35 | 70.0 (15) | 125.0 (25) | 0.27 | 4 | Metalaxyl ^13^C_6_ |
| Fungicide | Cyprodinil | 3.08 | 226.0 | 56 | 93.0 (33) | 108.0 (25) | 0.71 | 2 | Cyprodinil-d_5_ |
| Fungicide | Difenoconazole | 4.74 | 406.0 | 46 | 251.1 (25) | 111.1 (60) | 0.42 | 6 | Myclobutanil-d_4_ |
| Fungicide | Dimethomorph | 3.19;3.29 | 388.1 | 40 | 300.9 (20) | 165.0 (30) | 0.65;0.58 | 2 | Dimethomoprh-d_6_ |
| Fungicide | Fenamidone | 3.62 | 312.1 | 31 | 92.0 (25) | 236.1 (14) | 0.55 | 2 | Metalaxyl ^13^C_6_ |
| Fungicide | Fenhexamide | 3.71 | 302.1 | 41 | 97.2 (22) | 55.3 (38) | 0.47 | 2 | Fenhexamide-d_3_ |
| Fungicide | Fenpyrazamine | 3.86 | 332.0 | 30 | 216.0 (25) | 230.0 (20) | 1.45 | 2 | Fluopyram-d_4_ |
| Fungicide | Fludioxonil* | 3.41 | 247.0 | 50 | 180.0 (28) | 126.0 (35) | 0.78 | 20 | Metalaxyl ^13^C_6_ |
| Fungicide | Fluopicolide | 3.9 | 383.0 | 40 | 172.9 (20) | 365.0 (15) | 0.09 | 4 | Myclobutanil-d_4_ |
| Fungicide | Fluopyram | 3.96 | 397.0 | 20 | 173.0 (30) | 208.0 (20) | 0.73 | 2 | Fluopyram-d_4_ |
| Insecticide | Imidacloprid | 2.27 | 256.1 | 34 | 209.1 (15) | 175.1 (20) | 1.3 | 8 | Imidacloprid-d_4_ |
| Fungicide | Iprovalicarb | 3.55 | 321.1 | 28 | 119.0 (16) | 203.0 (10) | 0.37 | 2 | Metalaxyl ^13^C_6_ |
| Fungicide | Isofetamid | 4.54 | 360.0 | 15 | 125.0 (25) | 182.0 (15) | 0.57 | 2 | Fluopyram-d_4_ |
| Fungicide | Mandipropamid | 3.81 | 412.0 | 25 | 328.0 (16) | 125.0 (35) | 0.76 | 2 | Metalaxyl ^13^C_6_ |
| Fungicide | Metalaxyl | 2.88 | 280.1 | 26 | 220.1 (13) | 192.1 (17) | 0.51 | 2 | Metalaxyl ^13^C_6_ |
| Insecticide | Methiocarb | 3.3 | 226.0 | 28 | 169.0 (10) | 121.0 (22) | 0.56 | 2 | Metalaxyl ^13^C_6_ |
| Insecticide | Methoxyfenozide | 4.02 | 369.1 | 20 | 149.0 (20) | 313.2 (8) | 0.28 | 20 | Metalaxyl ^13^C_6_ |
| Fungicide | Metrafenone | 5.23 | 409.0 | 28 | 209.0 (14) | 227.0 (16) | 0.47 | 2 | Metalaxyl ^13^C_6_ |
| Fungicide | Myclobutanil | 3.59 | 289.1 | 34 | 70.2 (18) | 125.1 (32) | 0.26 | 2 | Myclobutanil-d_4_ |
| Fungicide | Penconazole | 4.05 | 284.0 | 34 | 70.1 (16) | 159.0 (34) | 0.43 | 2 | Myclobutanil-d_4_ |
| Fungicide | Propiconazole | 4.3 | 342.0 | 46 | 69.0 (22) | 159.0 (34) | 0.54 | 2 | Myclobutanil-d_4_ |
| Fungicide | Proquinazid | 6.02 | 373.0 | 25 | 331.0 (15) | 289.0 (25) | 0.59 | 2 | Fluopyram-d_4_ |
| Fungicide | Pyraclostrobin | 4.96 | 388.1 | 31 | 193.9 (12) | 163.0 (25) | 0.65 | 2 | Myclobutanil-d_4_ |
| Fungicide | Pyrimethanil | 2.68 | 200.0 | 51 | 82.0 (24) | 107.0 (24) | 1.52 | 2 | Pyrimethanil-d_5_ |
| Fungicide | Pyriofenone | 5.04 | 366.0 | 35 | 184.0 (25) | 209.0 (25) | 0.53 | 4 | Fluopyram-d_4_ |
| Fungicide | Quinoxyfen | 5.17 | 308.0 | 61 | 197.0 (32) | 161.9 (44) | 0.69 | 2 | Myclobutanil-d_4_ |
| Fungicide | Spiroxamine | 2.81 | 298.0 | 35 | 144.0 (20) | 100.0 (30) | 0.69 | 2 | Metalaxyl ^13^C_6_ |
| Fungicide | Tebuconazole | 3.89 | 308.0 | 40 | 70.1 (22) | 125.0 (40) | 0.07 | 2 | Tebuconazol-d_9_ |
| Insecticide | Tebufenozide | 4.52 | 353.1 | 19 | 133.0 (20) | 297.1 (8) | 0.2 | 4 | Tebuconazol-d_9_ |
| Fungicide | Tetraconazole | 3.85 | 371.9 | 41 | 159.0(30) | 70.1(20) | 0.98 | 2 | Tebuconazol-d_9_ |
| Insecticide | Thiamethoxam | 2.13 | 292.0 | 28 | 211.2 (12) | 132.0 (22) | 0.81 | 2 | Thiametoxam-d_4_ |
| Fungicide | Thiophanate methyl | 2.61 | 343.0 | 28 | 151.0 (46) | 93.0 (19) | 0.01 | 5 | Thiophanate methyl-d_6_ |
| Fungicide | Trifloxystrobin | 5.35 | 409.0 | 34 | 186.0 (16) | 145.0 (40) | 0.51 | 0.5 | Myclobutanil-d_4_ |
| Fungicide | Zoxamide | 4.83 | 336.0 | 38 | 187.1 (25) | 159.0 (38) | 0.55 | 0.5 | Myclobutanil-d_4_ |
|  | * ESI(-) |  |  |  |  |  |  |  |  |

Table S-1 cont. Determination conditions of isotopically labelled compounds added to samples as surrogate standards.

| **Compound** | **Ret. time (min)** | **Ion precursor** | **Cone voltage (V)** | **Q1 m/z (EC. eV)** | **Q2 m/z (EC. eV)** | **Ratio** **(Q2/Q1)** |
| --- | --- | --- | --- | --- | --- | --- |
| Acetamiprid-d_3_ | 2.32 | 226.1 | 34 | 126.0 (20) | 56.1 (15) | 0.99 |
| Boscalid-d_4_ | 3.73 | 347.0 | 41 | 140.0 (20) | 311.0 (20) | 2.1 |
| Carbendazim-d_3_ | 1.92 | 195.1 | 33 | 160.0 (18) | 132.0 (33) | 0.14 |
| Clothianidin-d_3_ | 2.21 | 253.0 | 20 | 172.0 (10) | 132.0 (15) | 0.64 |
| Cyprodinil-d_5_ | 3.05 | 231.0 | 56 | 93.0 (33) | 108.0 (25) | 0.47 |
| Dimethomoprh-d_6_ | 3.16; 3.27 | 394.2 | 40 | 307.1 (20) | 171.1 (30) | 0.57 |
| Fenhexamide-d_3_ | 3.71 | 305.0 | 50 | 100.0 (20) | 55.0 (30) | 0.5 |
| Fludioxonil ^13^C_2_* | 3.41 | 249.0 | 50 | 153.0 (30) | 128.0 (35) | 0.23 |
| Fluopyram-d_4_ | 3.95 | 401.0 | 20 | 177.0 (30) | 208.0 (20) | 0.8 |
| Imidacloprid-d_4_ | 2.26 | 260.1 | 34 | 213.1 (15) | 179.1 (20) | 1.2 |
| Metalaxyl ^13^C_6_ | 2.87 | 286.1 | 26 | 226.1 (13) | 198.1 (17) | 0.59 |
| Myclobutanil-d_4_ | 3.58 | 293.0 | 34 | 70.0 (18) | 129.0 (32) | 0.21 |
| Pyrimethanil-d_5_ | 2.66 | 205.0 | 51 | 107.0 (24) | 82.0 (24) | 0.69 |
| Tebuconazol-d_9_ | 3.85 | 317.0 | 40 | 70.1 (22) | 125.0 (40) | 0.07 |
| Thiametoxam-d_4_ | 2.13 | 296.0 | 20 | 215.0 (10) | 132.0 (20) | 0.44 |
| Thiophanate methyl-d_6_ | 2.6 | 349.0 | 21 | 151.0 (20) | 93.0 (50) | 0.14 |
| * ESI(-) |  |  |  |  |  |  |


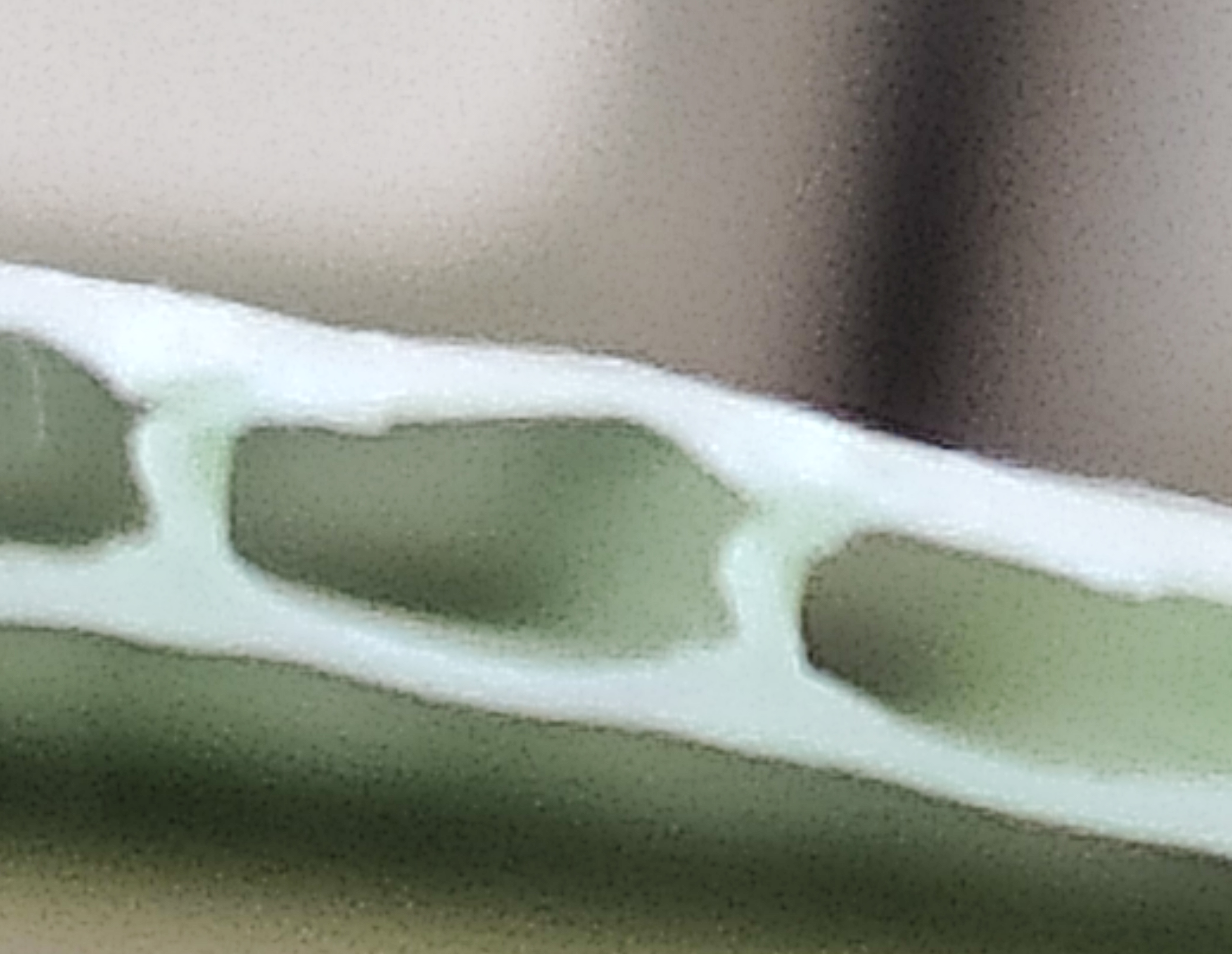


**V-10**

**a = inner sheet**

**b**

**c**

**d = outer sheet**

**1,8 mm**

Fig. S-1. Top: cross-section of V-10 shelter. Middle: normalized FTIR-ATR spectra recorded at different points of V-10 (marked in the cross section). Bottom: corresponding values ​​of the carbonyl index.

Fig. S-2. Normalized IR spectra corresponding to the outer face of tree shelters used for different times in different locations and applications.

Fig. S-3. Appearance and colour differences measured in the inner (I) and outer (O) surfaces of tree shelters used for 4 and 10 years. The 0a shelter was used as reference.

Fig. S-4. TG curves corresponding to homogenized sheets of shelters used for different times in different locations and applications.

Fig. S-5. Second heating DSC curves corresponding to homogenized sheets of two reference (unused) tree shelters.

Fig. S-6. DSC cooling curves (exo up) corresponding to shelters used for 4 (left) and 10 (right) years in vineyard plantations.

Table S-2. Melting and crystallization temperature, melting enthalpy and crystallinity (second heating scan) of homogenized specimens.

| **Sample** | **T_m_ (ºC)** | **T_c_ (ºC)** | **ΔH_m_(W/g)** | **X (%)** |
| --- | --- | --- | --- | --- |
| 0a | 168,3 | 129,0 | 74.6 | 36 |
| P-1 | 165,5 | 126,5 | 70.6 | 34 |
| V-4 | 163,5 | 122,2 | 84.3 | 40 |
| F-5 | 166,5 | 125,4 | 74.8 | 36 |
| V-10 | 163,8 | 126,2 | 71.6 | 35 |
| V-12 | 163,3 | 125,5 | 75.1 | 35 |
| V-15 | 164,3 | 124,1 | 77.8 | 38 |

Fig. S-7. DSC curves (cooling scan) corresponding to homogenized sheets.

Fig. S-8. OIT DSC curves of homogenized sheets of shelters.

Fig. S-9. Tensile properties of used shelters, measured in homogenized sheets.

Table S-3. Concentrations of fungicide residues determined in sample code V-12. Average values (ng g^-1^) for duplicate analysis.

| Compound | Concentration (ng g^-1^) |
| --- | --- |
| Benalaxyl  Cyprodinil  Difenoconazol  Dimethomorph  Fludioxonil  Metalaxyl  Myclobutanil  Penconazol  Proquinazid  Zoxamide | 5.4  290.0  8.8  148.0  60.8  69.6  24.4  37.6  21.2  68.0 |
